# *Ehrlichia chaffeensis* Tandem Repeat Effector Targets Differentially Influence Infection

**DOI:** 10.3389/fcimb.2017.00178

**Published:** 2017-05-12

**Authors:** Tian Luo, Paige S. Dunphy, Jere W. McBride

**Affiliations:** ^1^Department of Pathology, University of Texas Medical BranchGalveston, TX, USA; ^2^Department of Microbiology and Immunology, University of Texas Medical BranchGalveston, TX, USA; ^3^Center for Biodefense and Emerging Infectious Diseases, University of Texas Medical BranchGalveston, TX, USA; ^4^Sealy Center for Vaccine Development, University of Texas Medical BranchGalveston, TX, USA; ^5^Institute for Human Infections and Immunity, University of Texas Medical BranchGalveston, TX, USA

**Keywords:** *Ehrlichia chaffeensis*, tandem repeat protein, effector-host interaction, infection, cell signaling, vesicle trafficking, transcriptional regulation, posttranslational modification

## Abstract

*Ehrlichia chaffeensis* infects mononuclear phagocytes and survives intracellularly by exploiting host cell processes to evade host defenses. The mechanisms involved are not fully defined, but appear to rely largely on a subset of tandem repeat proteins (TRP) effectors. *E. chaffeensis* TRPs are type 1 secreted effectors that interact with a functionally diverse group of host cell targets associated with various biological processes. In this study, we investigated the influence of TRP host target proteins on ehrlichial infection by RNA interference. In total, 138 TRP-interacting host proteins identified by yeast two-hybrid were targeted by siRNA and the infection level determined by real-time qPCR. Knockdown of 124 (89%) TRP target proteins had significant influence on infection either by inhibiting (85%) or promoting (15%) ehrlichial infection. Notably, knockdown of 18 host proteins which interacted with TRP120 promoted the infection, suggesting that these targets may be degraded to promote infection. Host proteins that interact with TRPs are involved in cellular processes, including cell signaling, vesicle trafficking and intracellular transport, transcriptional regulation, metabolism, protein posttranslational modification, and apoptosis. Selected host targets were examined by immunofluorescent microscopy during infection and were found to localize with the morulae, or in the host cell cytoplasm adjacent to morulae. This study confirms that the majority of host proteins known to interact with TRP effectors influence infection and further extends the current knowledge that *E. chaffeensis* TRPs participate in a complex array of host protein interactions in order to reprogram the host cell and promote intracellular survival.

## Introduction

*Ehrlichia chaffeensis* is an obligately intracellular bacterium and the etiologic agent of the emerging life-threatening human zoonosis, human monocytotropic ehrlichiosis (HME) (Paddock and Childs, 2003). *E. chaffeensis* selectively infects mononuclear phagocytes and resides in endosome-like membrane-bound vacuoles where it replicates and evades innate host defenses (Paddock and Childs, 2003). The mechanisms by which *E. chaffeensis* enters the host cell, avoids destruction, and establishes persistent infection are not well-understood, but functionally relevant host-pathogen interactions are essential for reprogramming the host cell defense mechanisms. This molecular strategy involves type 1 secreted tandem repeat protein (TRP) effectors (Lina et al., 2016b).

*E. chaffeensis* TRPs are major immunoreactive proteins that elicit strong host antibody responses during infection. The tandem repeat (TR) domains in TRP120, TRP47, and TRP32 are acidic, serine-rich, and contain protective species-specific epitopes (Doyle et al., 2006; Luo et al., 2008, 2009; Kuriakose et al., 2012). TRP120 and TRP47 are differentially expressed by infectious dense cored cells (DC), while TRP32 is expressed by both DCs and replicating reticulate cells (RC) (Popov et al., 2000; Doyle et al., 2006; Luo et al., 2008). Consistent with type 1 secretion (T1S) signals identified in the C-terminal domains of TRPs, TRPs have been experimentally identified as T1S system substrates through studies using a heterologous T1S apparatus of *Escherichia coli* (Wakeel et al., 2011).

In order to identify *Ehrlichia*-host interactions and help define the complex mechanisms by which *E. chaffeensis* modulates host cells, multiple studies using the yeast two-hybrid (Y2H) approach have been performed to better understand molecular host-pathogen interactions involving TRPs. TRP120, TRP47, and TRP32 have been shown to interact with a diverse network of host proteins involved in many host cellular processes including cell signaling, vesicle trafficking and intracellular transport, transcriptional regulation, metabolism, posttranslational modification and apoptosis, indicating the important roles of TRPs in reprogramming the host cell (Wakeel et al., 2009; Luo et al., 2011; Luo and McBride, 2012).

TRPs are modified by multiple host posttranslational modification pathways, including SUMOylation, ubiquitination and phosphorylation, which appear to mediate functional interactions and extend the number and diversity of interactions with host targets, as well as localization to various subcellular locations, including the nucleus (Wakeel et al., 2010; Dunphy et al., 2014). TRP120 is modified by SUMO at a canonical consensus SUMO conjugation motif located in the C-terminal domain, which has been further confirmed using a high-density microfluidic peptide array (Zhu et al., 2016). TRP120 conjugation with SUMO mediates interactions with host protein targets, and inhibition of the host SUMO pathway significantly decreases interaction between TRP120 and host protein targets, resulting in decreased ehrlichial intracellular survival (Dunphy et al., 2014). TRP120 also interacts with components of the ubiquitin pathways, including the E3 ligases, KLHL12 and FBXW7 as well as ubiquitin (Ub) isoforms UBB and UBC, which suggests TRP120 is a target of Ub conjugation (Luo et al., 2011). TRP47 is phosphorylated and interacts with the Src family tyrosine kinase, Fyn, which may be involved in the tyrosine phosphorylation of TRP47 (Wakeel et al., 2009, 2010). TRPs also contain many additional predicted phosphorylation sites; however, it is not clear which protein kinases are involved and how the phosphorylation affects TRP function or interactions with the host cell.

We have demonstrated the influence of selected TRP120 or TRP32-interacting host proteins on ehrlichial infection by RNA interference (Luo and McBride, 2012; Luo et al., 2016); however, a comprehensive analysis of all TRP-host interactions has not been performed. In this study, we extend the role of TRP-host interactions by investigating the influence of 138 TRP120, TRP47, and TRP32 interacting host target proteins on ehrlichial infection by RNA interference. We directly demonstrate that *E. chaffeensis* exploits the host cells through complex TRP interactions with a large and diverse array of host targets to promote intracellular survival.

## Materials and methods

### Cell culture and cultivation of *E. chaffeensis*

Human monocytic leukemia cells (THP-1, from ATCC) were propagated in RPMI medium 1640 with L-glutamine and 25 mM HEPES buffer (Invitrogen, Carlsbad, Invitrogen), supplemented with 1 mM sodium pyruvate, 2.5 g/L D-(+)-glucose (Sigma, St. Louis, MO), and 10% fetal bovine serum (HyClone, Logan, UT). *E. chaffeensis* (Arkansas strain) was cultivated in THP-1 cells as previously described (Kuriakose et al., 2011).

### siRNAs and antibodies

All specific siRNAs were MISSION esiRNA from Sigma, which are endoribonuclease-prepared siRNA pools comprised of a heterogeneous mixture of siRNAs that all target the same mRNA sequence. These multiple silencing triggers lead to highly specific and effective gene silencing (guaranteed > 70% knockdown at the mRNA level) with lower off-target effects than single or pooled siRNAs. Targeting sequences of all siRNAs can be found on Sigma website. The control siRNA was ON-TARGET*plus* non-targeting siRNA from GE Healthcare Dharmacon (Lafayette, CO), which was designed to leverage seed-region optimization and patented modification patterns to have fewer off-targets than traditionally designed, unmodified negative control siRNAs. Thus, changes in mRNA or protein levels in cells treated with the control reflect a baseline cellular response that can be compared to the levels in cells treated with target-specific siRNA. Alexa Fluor 488-labeled negative siRNA was from Qiagen (Germantown, MD). Rabbit and mouse anti-TRP32, TRP47, or TRP120 antibodies have been described previously (Kuriakose et al., 2012). Other antibodies used in this study were mouse anti-human FBXW7 (R&D Systems, Minneapolis, MN), α-tubulin and PTPN2 (Santa Cruz, Dallas, TX), and rabbit anti-human CD14 (Abgent, San Diego, CA), EIF3A, ERA1, KARS, LMAN1, and STAT6 (Proteintech, Rosemont, IL), FYN and SLC43A3 (Santa Cruz), IRF2BP2 and SEPX1 (Pierce, Rockford, IL), KDM6B (Novus, Littleton, CO), and RC3H1 (Sigma).

### RNA interference

THP-1 cells (1 × 10^5^/well on a 96-well plate) were transfected with 5 pmol siRNA using Lipofectamine 3000 (Invitrogen) according to the manufacturer's protocol. An Alexa Fluor 488-labeled negative siRNA was used as a control to monitor transfection efficiency. At 1 day posttransfection, the cells were synchronously infected by cell-free *E. chaffeensis* at a MOI of ~50, and collected at 1 and 2 days postinfection (p.i.) for Western blot and quantitative PCR (qPCR) to determine knockdown levels and infection status, respectively. The experiment of each siRNA was repeated for at least three times. Infectious dense-cored (DC) ehrlichiae enter the host cell and transform into large replicating reticulate cells (RC). At 1 day p.i. ehrlichiae are represented predominantly by RC and RC ehrlichiae replicate during the next 2 days p.i. before maturing into DC at 3 days. Thus, infected cells were not collected at 3 days p.i. since some cells started to collapse and release *Ehrlichia*.

### Quantification of *E. chaffeensis* by qPCR

THP-1 cells were pelleted, washed by PBS, lysed in SideStep lysis and stabilization buffer (Agilent, Santa Clara, CA) for 10 min at room temperature with shaking, and analyzed for bacterial load using real-time qPCR. Amplification of the integral ehrlichial disulfide bond formation protein (*dsb*) gene and human glyceraldehyde-3-phosphate dehydrogenase (*gapdh*) gene was performed separately using Brilliant II SYBR Green mastermix (Agilent), 200 nM forward primers (*dsb*: 5′-gctgctccaccaataaatgtatccct-3′; *gapdh*: 5′-ggagtccactggcgtcttcac-3′) and 200 nM reverse primers (*dsb*: 5′-gtttcattagccaagaattccgacact-3′; *gapdh*: 5′-gaggcattgctgatgatcttgag-3′). The qPCR thermal cycling protocol (denaturation at 95°C 10 min, then 40 cycles of 95°C 30 s, 58°C 30 s, 72°C 30 s) was performed on the 7900HT fast real-time PCR system (Applied Biosystems, Foster City, CA). The fold change of *dsb* copy number relative to the control was normalized to qPCR-detected levels of the host *gapdh* gene.

### Western immunoblot

The THP-1 cell lysates were prepared using CytoBuster protein extraction reagent (Novagen/EMD, Gibbstown, NJ), separated by sodium dodecyl sulfate-polyacrylamide gel electrophoresis (SDS-PAGE) and transferred to nitrocellulose membrane. Western immunoblot was performed with horseradish peroxidase-labeled goat anti-rabbit, or mouse IgG (heavy and light chains) conjugate (Kirkegaard & Perry Laboratories, Gaithersburg, MD) and SuperSignal West Dura chemiluminescent substrate (Thermo Scientific).

### Immunofluorescence microscopy

Uninfected or *E. chaffeensis-*infected THP-1 cells at 2 days p.i. were collected, and the indirect immunofluorescent antibody assay was performed as previously described (Luo et al., 2011). Fluorescence images were obtained using a BX61 epifluorescence microscope (Olympus, Japan) and the Slidebook 5.0 software (Intelligent Imaging Innovations, Denver, CO).

### Statistics

The statistical differences between experimental groups were assessed with the two-tailed Student's *t*-test, and significance was indicated by a *P* < 0.05.

## Results

### Demonstration of knockdown of protein expression by western blot analysis

**Figure 1:**
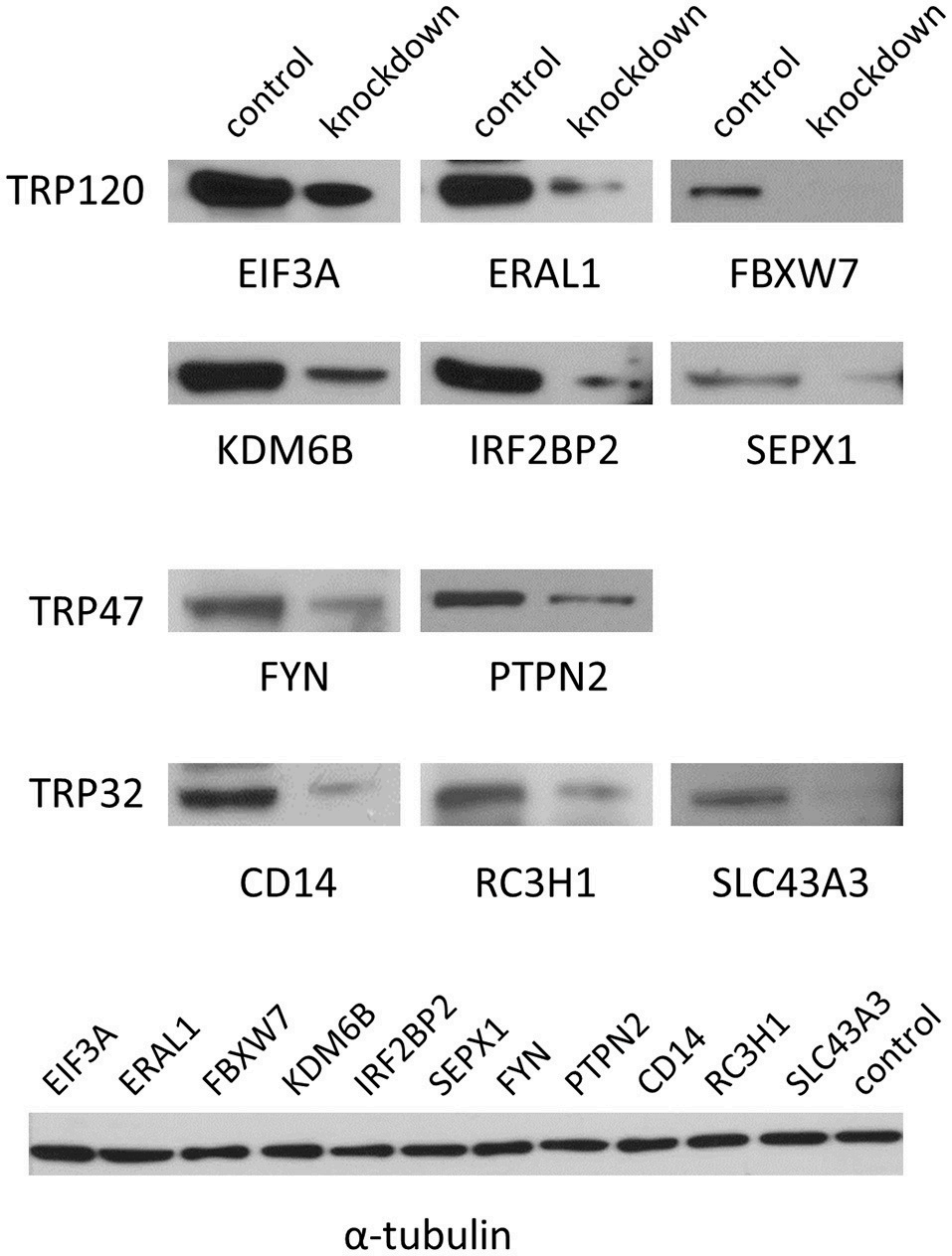
**Verification of knockdown of TRP-interacting host proteins by Western blot**. THP-1 cells were transfected with each specific or control siRNA and 2 days posttransfection Western blots were performed to determine knockdown efficiency. The α-tubulin was visualized as a control of Western blot.

To verify the knockdown of gene expression by siRNA, 11 target proteins of high priority for the *Ehrlichia*-host interaction study were detected at 2 days posttransfection by Western blot, including six TRP120-interacting proteins eukaryotic translation initiation factor 3 subunit A (EIF3A), Era-like 12S mitochondrial rRNA chaperone 1 (ERA1), F-box and WD repeat domain containing 7 (FBXW7), lysine (K)-specific demethylase 6B (KDM6B), interferon regulatory factor 2 binding protein (IRF2BP2), and selenoprotein X 1 (SEPX1), two TRP47-interacting proteins FYN proto-oncogene Src family tyrosine kinase (FYN) and protein tyrosine phosphatase non-receptor type 2 (PTPN2), and three TRP32-interacting proteins CD14 molecule (CD14), ring finger and CCCH-type domain 1 (RC3H1) and solute carrier family 43 member 3 (SLC43A3). The expression of all these proteins was substantially reduced in specific siRNA-transfected cells, respectively, compared with the unrelated control siRNA-transfected cells, indicating the successful knockdown of gene expression and good efficiency of siRNAs (Figure 1).

### Impact of knockdown of TRP120-interacting proteins on *E. chaffeensis* survival in host cells

**Figure 2:**
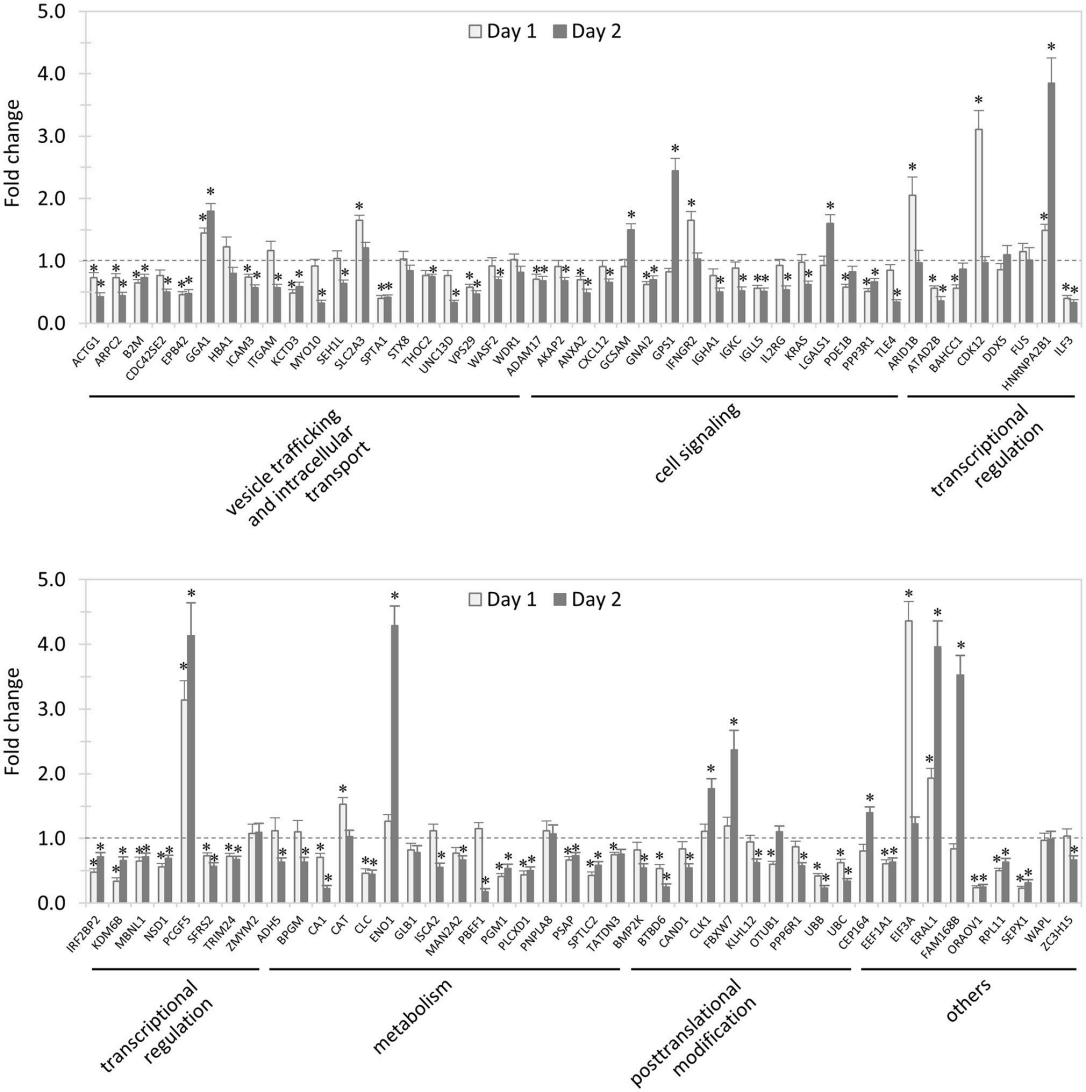
**Influence of knockdown of TRP120-interacting proteins on ***Ehrlichia*** infection**. THP-1 cells were transfected with target or control siRNA, and then infected by *E. chaffeensis*. Infection status was determined by qPCR at 1 and 2 days postinfection, compared to control scrambled siRNA-transfected cells. *E. chaffeensis dsb* gene copy numbers were normalized to host *gapdh* gene. Results were from three independent experiments, and the values were means ± standard deviations (^*^P < 0.05, significantly different from control infection).

In total, 89 siRNAs available on the market were used to target 89 TRP120-interacting host proteins respectively, and then the impact of each siRNA on ehrlichial infection was assessed by qPCR at 1 and 2 days (Figure 2) p.i. Overall, knockdown of 80 (90% of 89) TRP120 target proteins had significant influence on ehrlichial infection, while only 9 (10%) proteins did not. Knockdown of 62 (70%) proteins inhibited ehrlichial infection, whereas knockdown of 18 (20%) proteins promoted ehrlichial infection. At 1 day p.i., the decrease of 39 (44%) proteins decreased *Ehrlichia* infection significantly, whereas the decrease of 11 (12%) proteins increased *Ehrlichia* infection significantly. At 2 days p.i., the decrease of 58 (65%) proteins decreased *Ehrlichia* infection significantly, whereas the decrease of 12 (14%) proteins increased *Ehrlichia* infection significantly (Table 1). The results indicated that most TRP120-interacting host proteins influence *E. chaffeensis* infection, and most TRP120 target proteins promote *Ehrlichia* infection. Knockdown of some targets largely associated with transcriptional regulation (day 1) increased infection, suggesting that these are downregulated or degraded by *Ehrlichia* to promote infection.

**Table 1:** **Impact of TRP120-interacting protein knockdown on ***E. chaffeensis*** survival in host cells**.

	**Number (percentage) of genes whose knockdown had an impact on *E. chaffeensis* infection**			**Total**
	**Inhibition**	**Promotion**	**No significant change**	
By day				
Day 1	39 (44%)	11 (12%)	39 (44%)	89
Day 2	58 (65%)	12 (14%)	19 (21%)	
Overall	62 (70%)	18 (20%)	9 (10%)	
By category of function				89
Vesicle trafficking and intracellular transport	15	2	3	20
Cell signaling	13	4	0	17
Transcriptional regulation	9	4	3	16
Metabolism	12	2	2	16
Posttranslational protein modification	8	2	0	10
Other miscellaneous	5	4	1	10

TRP120-interacting proteins include host targets involved in vesicle trafficking and intracellular transport, cell signaling, transcriptional regulation, posttranslational modification, metabolism, and others. In each category, knockdown of most or all target proteins had significant influence on ehrlichial infection, and the knockdown of most proteins caused inhibition of infection, consistent with the overall impact of TRP120-interacting proteins (Table 1). The results indicated that TRP120 has moonlighting functions through interactions with diverse targets to modulate important host cellular processes to facilitate *Ehrlichia* survival.

### Impact of knockdown of TRP47-interacting proteins on *E. chaffeensis* survival in host cells

**Figure 3:**
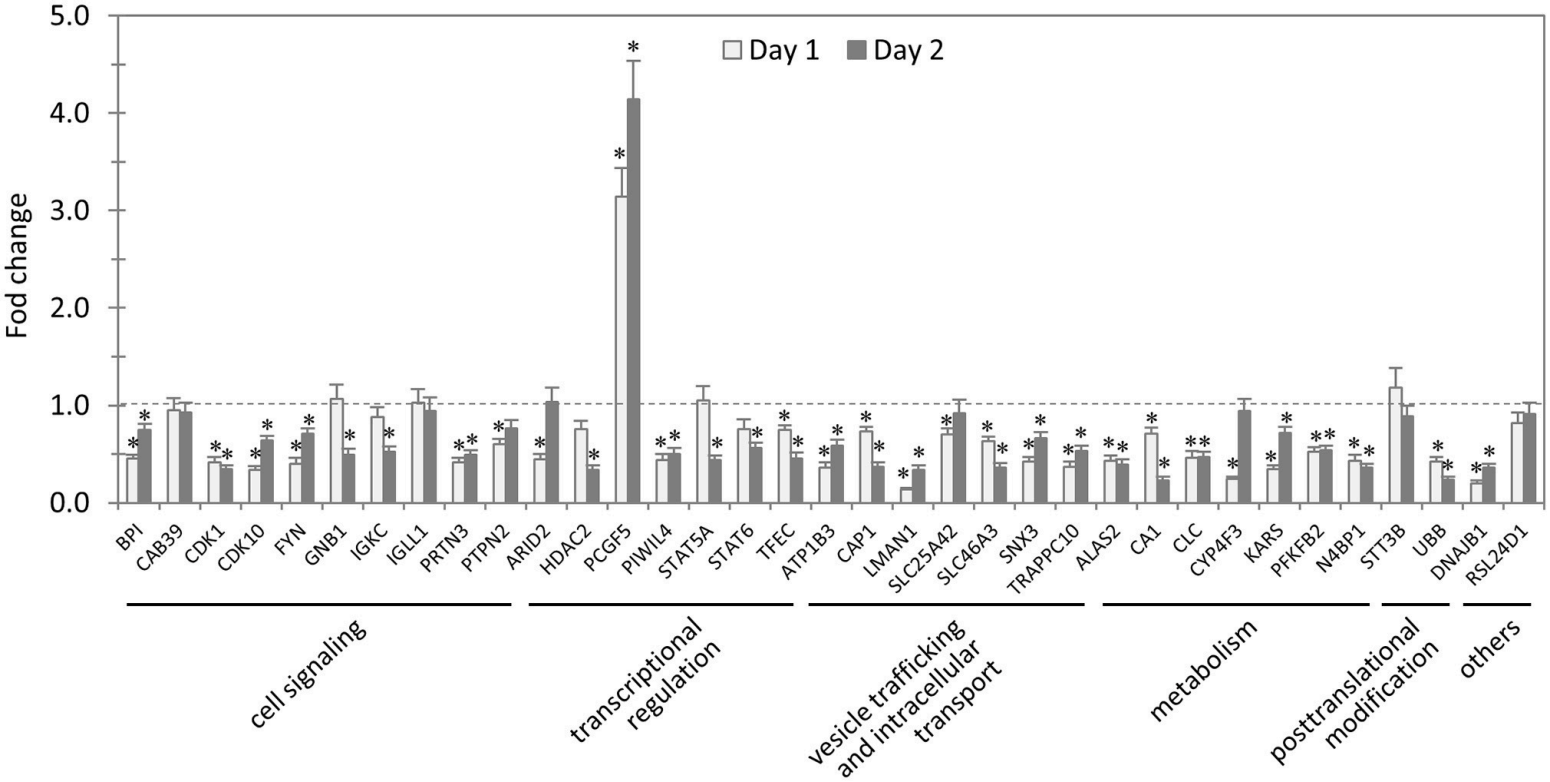
**Influence of knockdown of TRP47-interacting proteins on ***Ehrlichia*** infection**. THP-1 cells were transfected with target or control siRNA, and then infected by *E. chaffeensis*. Bacterial number changes were determined by qPCR at 1 and 2 days postinfection, compared to control scrambled siRNA-transfected cells. *E. chaffeensis dsb* gene copy numbers were normalized to host *gapdh* gene. Results were from three independent experiments, and the values were means ± standard deviations (^*^P < 0.05, significantly different from control infection).

In total, 35 siRNAs available on the market were used to target 35 TRP47-interacting host proteins respectively, and then the impact of each siRNA on ehrlichial infection was assessed by qPCR at 1 and 2 days p.i. (Figure 3). Overall, knockdown of 31 (89% of 35) TRP47 target proteins had significant influence on ehrlichial infection, while only 4 (11%) proteins did not. Knockdown of 30 (86%) proteins inhibited ehrlichial infection, whereas knockdown of only 1 (3%) protein (polycomb group ring finger 5 [PCGF5]) that also interacted with TRP120 promoted ehrlichial infection. At both 1 and 2 days p.i., only PCGF5 knockdown increased *E. chaffeensis* infection significantly, but the knockdown of 25 (71%) proteins decreased the infection significantly at 1 day p.i., and the knockdown of 26 (74%) proteins negatively affected infection significantly at 2 days p.i. (Table 2). Therefore, consistent with TRP120-interacting proteins, most TRP47-interacting host proteins play a role in *E. chaffeensis* survival by either inhibiting or promoting the infection; moreover, most TRP47-interacting proteins appear to promote rather than inhibit *Ehrlichia* infection.

**Table 2:** **Impact of TRP47-interacting protein knockdown on ***E. chaffeensis*** survival in host cells**.

	**Number (percentage) of genes whose knockdown had an impact on *E. chaffeensis* infection**			**Total**
	**Inhibition**	**Promotion**	**No significant change**	
By day				
Day 1	25 (71%)	1 (3%)	9 (26%)	35
Day 2	26 (74%)	1 (3%)	8 (23%)	
Overall	30 (86%)	1 (3%)	4 (11%)	
By category of function				35
Cell signaling	8	0	2	10
Vesicle trafficking and intracellular transport	7	0	0	7
Transcriptional regulation	6	1	0	7
Metabolism	6	0	0	6
Posttranslational protein modification	2	0	1	3
Others	1	0	1	2

TRP47-interacting proteins include host targets involved in cell signaling, transcriptional regulation, vesicle trafficking and intracellular transport, metabolism and posttranslational modification, consistent with TRP120-interacting proteins. In each category, knockdown of most or all target proteins had significant influence on ehrlichial infection, and the knockdown of majority of these protein caused inhibition of infection, consistent with the overall influence of TRP47-interacting proteins (Table 2). The results indicated that TRP47 has moonlighting functions through interactions with diverse targets to modulate important host cellular processes to facilitate *Ehrlichia* survival.

### Impact of knockdown of TRP32-interacting proteins on *E. chaffeensis* survival in host cells

**Figure 4:**
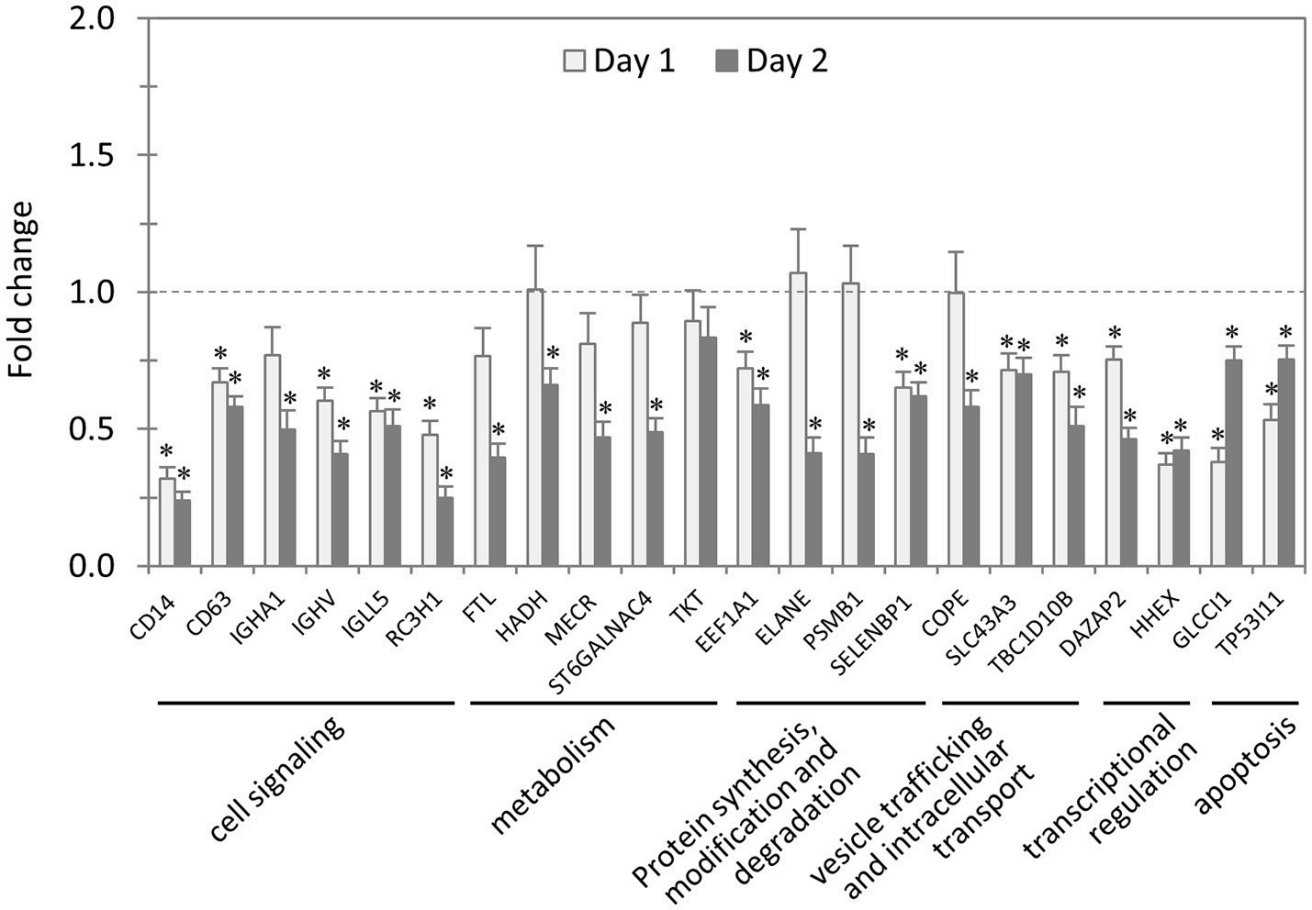
**Influence of knockdown of TRP32-interacting proteins on ***Ehrlichia*** infection**. THP-1 cells were transfected with target or control siRNA, and then infected by *E. chaffeensis*. Bacterial number changes were determined by qPCR at 1 and 2 days postinfection, compared to control scrambled siRNA-transfected cells. *E. chaffeensis dsb* gene copy numbers were normalized to host *gapdh* gene. Results were from three independent experiments, and the values were means ± standard deviations (^*^P < 0.05, significantly different from control infection).

In total, 22 siRNAs were used to target 22 TRP32-interacting host proteins respectively, and then the impact of each siRNA on ehrlichial infection was assessed by qPCR (Figure 4). Overall, knockdown of 21 (95% of 22) TRP32 target proteins significantly reduced ehrlichial infection, while knockdown of only 1 (5%) protein (transketolase [TKT]) did not have significant impact on infection. None of TRP32-interacting protein knockdowns promoted ehrlichial infection. At 1 day p.i., the decrease in 13 (59%) proteins had a significant negative impact on *Ehrlichia* infection, and at 2 days p.i., the decrease in 21 (95%) proteins had a significant negative impact on *Ehrlichia* infection (Table 3). The results indicated that almost all TRP32-interacting proteins play a role in *E. chaffeensis* survival by promoting the infection.

**Table 3:** **Impact of TRP32-interacting protein knockdown on ***E. chaffeensis*** survival in host cells**.

	**Number (percentage) of genes whose knockdown had an impact on *E. chaffeensis* infection**			**Total**
	**Inhibition**	**Promotion**	**No significant change**	
By day				
Day 1	13 (59%)	0	9 (41%)	22
Day 2	21 (95%)	0	1 (5%)	
Overall	21 (95%)	0	1 (5%)	
By category of function				22
Cell signaling	6	0	0	6
Metabolism	4	0	1	5
Protein synthesis, modification and degradation	4	0	0	4
Vesicle trafficking and intracellular transport	3	0	0	3
Transcriptional regulation	2	0	0	2
Apoptosis	2	0	0	2

TRP32-interacting proteins include host targets involved in cell signaling, metabolism, protein synthesis, modification and degradation, vesicle trafficking and intracellular transport, transcriptional regulation, and apoptosis, similar to TRP120/TRP47-interacting proteins. In each category, knockdown of most or all target proteins significantly reduced *Ehrlichia* infection, consistent with the overall influence of TRP32-interacting proteins (Table 3). The results indicated that TRP32 plays multiple roles in reprogramming important host cellular processes.

### Overall impact of knockdown of TRP-interacting proteins on *E. chaffeensis* survival in host cells

By Y2H we have previously identified totally 150 host proteins interacting with *E. chaffeensis* TRPs, including 98 proteins with TRP120, 38 proteins with TRP47, and 22 proteins with TRP32, while TRP120 shares 8 common targets with TRP47 and TRP32 (Wakeel et al., 2009; Luo et al., 2011; Luo and McBride, 2012). In this study, 138 (92% of 150) siRNAs were used to reduce the expression of corresponding genes respectively, including 89 siRNAs for TRP120 targets, 35 siRNAs for TRP47 targets, and 22 siRNAs for TRP32 targets. Overall, knockdown of 124 (89% of 138) TRP target proteins had significant influence on ehrlichial infection. Among 124 TRP target proteins, knockdown of 105 (85%) proteins inhibited ehrlichial infection, whereas knockdown of 19 (15%) proteins promoted ehrlichial infection. The inhibition or promotion of ehrlichial survival occurred at different stages of infection (1 or/and 2 days p.i.). For each TRP, target proteins were consistently involved in similar important host cell processes.

### TRP-expressing ehrlichiae colocalize with human target proteins in *E. chaffeensis*-infected THP-1 cells

**Figure 5:**
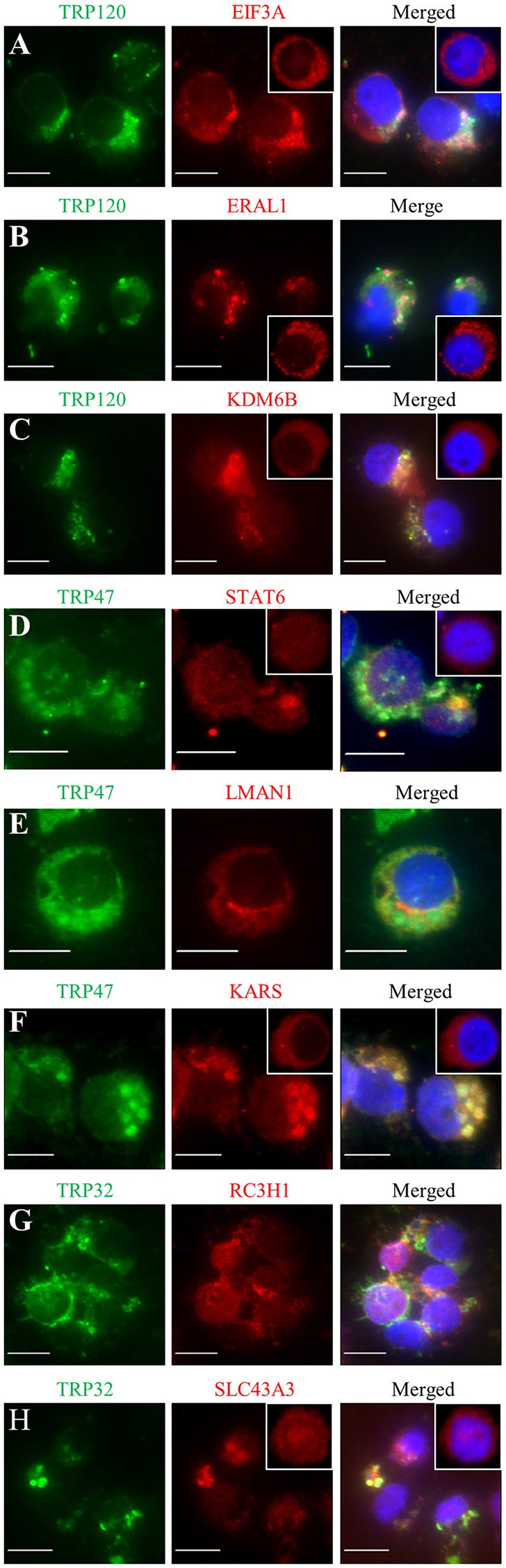
**Colocalization of TRP120, TRP47, and TRP32 with their interacting host proteins in ***E. chaffeensis***-infected THP-1 cells**. Fluorescence microscopy of infected (2 days postinfection) THP-1 cells stained with 4,6′-diamidino-2-phenylindole (blue, showing the nucleus), TRP antibody (green), and host protein antibody (red; panels **A–H**, respectively) show co-localization of *E. chaffeensis* TRP-labeled morulae with host protein. The insets (panels **A–D**,**F**,**H**) show the distribution of EIF3A, ERAL1, KDM6B, STAT6, KARS, and SLC43A3 in uninfected THP-1 cells. Fluorescence images were obtained using an Olympus BX61 epifluorescence microscope. Bar, 10 μm.

Previously, we have demonstrated the interactions of many identified target proteins with *E. chaffeensis* TRP120, TRP47, or TRP32 by multiple approaches (Wakeel et al., 2009; Luo et al., 2011; Luo and McBride, 2012). Here we examined host target proteins with well-defined and interesting functions and confirmed the interactions with *E. chaffeensis* TRPs by immunofluorescence assay. Eight target proteins were examined, including three TRP120-interacting proteins EIF3A, ERA1, and KDM6B, three TRP47-interacting proteins signal transducer and activator of transcription 6 (STAT6), lectin mannose binding 1 (LMAN1), and lysyl-tRNA synthetase (KARS), and two TRP32-interacting proteins RC3H1 and SLC43A3. Consistent with our previous reports, double-immunofluorescence labeling of *E. chaffeensis*-infected THP-1 cells revealed seven proteins EIF3A, ERA1, KDM6B, STAT6, KARS, RC3H1, and SLC43A3 exhibited strong colocalization with the morulae (Figures 5A–D,F–H), while LMAN1 localized in the host cell cytoplasm adjacent to the morula membrane (Figure 5E). Redistribution of TRP target proteins in *E. chaffeensis*-infected cells compared to uninfected cells was observed. For example, EIF3A, ERAL1, KDM6B, STAT6, KARS, and SLC43A3 were associated with morulae in infected THP-1 cells (Figures 5A–D,F–H), while in uninfected THP-1 cells, EIF3A, ERAL1, KDM6B, and KARS had diffused or punctate distribution mainly in the cytoplasm (Figures 5A–C,F, insets), and STAT6 and SLC43A3 were distributed throughout the whole cell (Figures 5D,H, insets).

## Discussion

**Figure 6:**
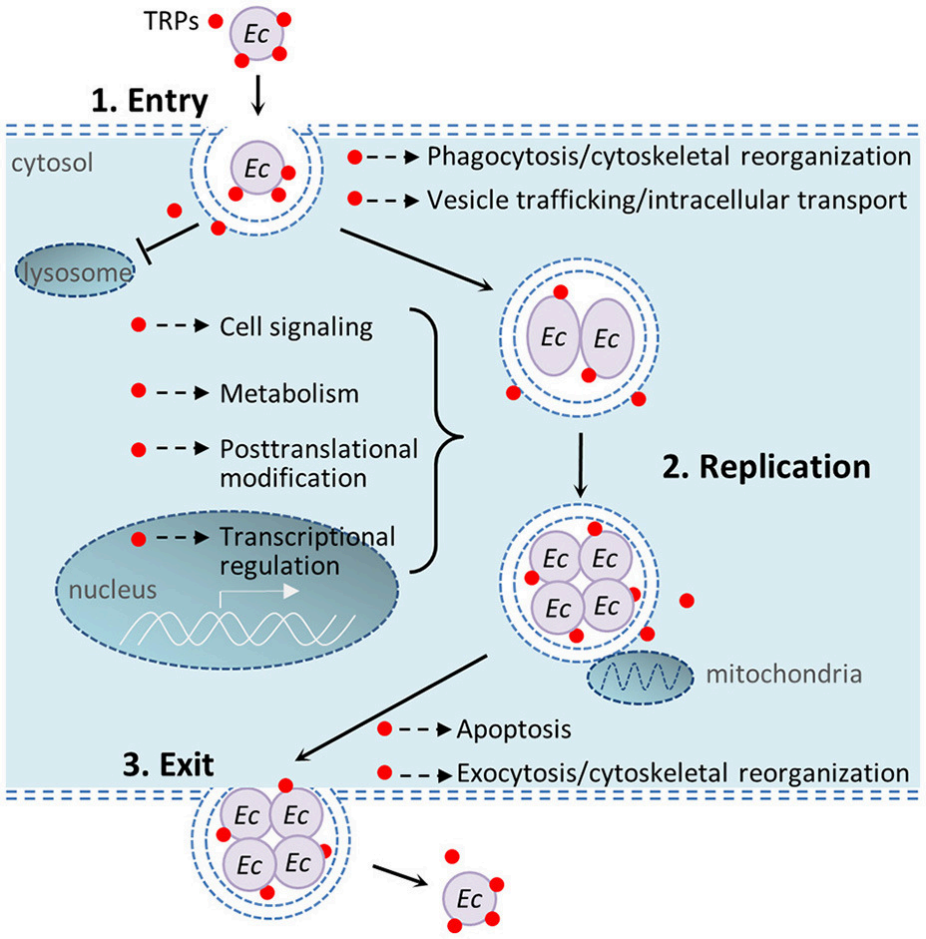
**Schematic diagram of important host cellular processes that ***E. chaffeensis*** tandem repeat proteins modulate during infection**. 1. Bacterial entry: *E. chaffeensis* binds and enters the host cell by phagocytosis. TRPs interact with host cytoskeletal proteins including actins and actin-related proteins, and also exploit host cell signaling (such as Wnt) to facilitate phagocytosis; TRPs also interact with host vesicular trafficking system to help establish an intracellular niche in a membrane-bound vacuole that does not undergo phagolysosomal fusion. 2. Bacterial replication: TRPs are secreted into the intramorular space and translocate into cytosol and nucleus of the host cell, where these effectors interact with diverse host proteins to modulate cell processes, including vesicle trafficking and intracellular transport, cell signaling, transcriptional regulation, posttranslational modification, metabolism, apoptosis, and others in order to avoid host defense system and permit intracellular replication. 3. Bacterial exit: TRPs interact with many apoptosis-associated proteins (some in mitochondria) to induce host cell apoptosis; TRPs also interact with host cytoskeletal proteins to facilitate exocytosis. Red dots show TRPs, including TRP120, TRP47, and TRP32. *Ec, E. chaffeensis*.

In recent years multiple studies from our laboratory have identified numerous *Ehrlichia*-host interactions and determined that *Ehrlichia* TRPs interact with a diverse network of host proteins involved in many host cellular processes including cell signaling, vesicle trafficking and intracellular transport, transcriptional regulation, metabolism, posttranslational modification, and apoptosis (Wakeel et al., 2009; Luo et al., 2011; Luo and McBride, 2012). Previous studies have helped understand the complex mechanisms by which *E. chaffeensis* modulates host cell processes. This study found that knockdown of host proteins that interact with *Ehrlichia* TRPs differentially influence intracellular survival. Figure 6 illustrates important host cellular processes that *E. chaffeensis* TRPs modulate during infection through interactions with host targets.

Notably, *E. chaffeensis* TRPs interact with numerous host proteins associated with cellular signaling pathways, including 17 proteins with TRP120, 10 proteins with TRP47, and six proteins with TRP32, suggesting that infection and replication of *E. chaffeensis* involves TRP exploitation of conserved cell signaling pathways, such as Wnt, Notch, mitogen-activated protein kinase/extracellular signal-regulated kinases (MAPK/ERK), phosphatidylinositol 3-kinase/Akt/mechanistic target of rapamycin (PI3K/Akt/mTOR), nuclear factor kappa B (NFκB), and cytokine/chemokine-mediated signaling (Table 4). Wnt and Notch are fundamental cell signaling pathways that play major roles in the regulation of gene expression, cell proliferation and differentiation, and embryonic development (Hayward et al., 2008). Five TRP120-interacting proteins including ADAM metallopeptidase domain 17 (ADAM17), annexin A2 (ANXA2), protein phosphatase 3 regulatory subunit B alpha (PPP3R1), transducin like enhancer of split 4 (TLE4), and G protein subunit beta 1 (GNB1) are signaling molecules of Wnt and Notch pathways. This is consistent with recent studies in which we have used different approaches to demonstrate that Wnt and Notch signaling pathways play important roles in ehrlichial infection, and TRPs mediate ehrlichial invasion and survival through activation and modulation of Wnt and Notch pathways (Luo et al., 2016; Lina et al., 2016a). In this study, we further demonstrated that knockdown of nearly all of TRP-interacting proteins involved in host cell signaling, except two TRP47-interacting proteins, calcium binding protein 39 (CAB39) and immunoglobulin lambda like polypeptide 1 (IGLL1), significantly influenced *E. chaffeensis* survival. These results support the importance of many conserved cellular signaling pathways in ehrlichial survival. For example, G-protein coupled receptor (GPCR) signaling involves a variety of ligands and stimuli and can regulate a highly interconnected network of signal transductions and cellular responses (Roth et al., 2015). We found that six TRP120-interacting proteins including ADAM17, A kinase anchor protein 2 (AKAP2), C-X-C motif chemokine ligand 12 (CXCL12), G protein subunit alpha i2 (GNAI2), G protein pathway suppressor 1 (GPS1), and phosphodiesterase 1B (PDE1B), and one TRP47-interacting protein GNB1 are involved in GPCR signaling. In addition, MAPK signaling is a conserved pathway involved in a variety of fundamental cellular processes (Cargnello and Roux, 2011). It has been reported that p38 MAPK/ERK1/2 are activated at the early stage of *E. chaffeensis* infection, but the activations are subsequently reduced in infected cells compared with those in uninfected cells (Lin and Rikihisa, 2004). We found that six TRP120-interacting proteins GNAI2, GPS1, immunoglobulin kappa constant (IGKC), interleukin 2 receptor subunit gamma (IL2RG), KRAS proto-oncogene GTPase (KRAS), and PPP3R1, four TRP47-interacting proteins cyclin dependent kinase 1 (CDK1), cyclin dependent kinase 10 (CDK10), FYN, and IGKC, and one TRP32-interacting protein, CD14, are involved in MAPK signaling. Knockdown of all these TRP target proteins significantly influenced infection, indicating that GPCR and MAPK signaling pathways are involved in ehrlichial survival. Based on these studies, Wnt, Notch, and MAPK as well as GPCR signaling pathway appear to be major targets of TRP exploitation. Further investigation is needed to understand the specific role of each TRP target and associated pathway(s) in ehrlichial pathobiology.

**Table 4:** *****E. chaffeensis*** TRP-interacting host proteins with primary function in cell signaling**.

**TRP**	**Host protein**		**Signaling pathway**
	**Symbol**	**Full name**	
TRP120	ADAM17	ADAM metallopeptidase domain 17	GPCR, Notch, Hedgehog, EGFR, TGFβ, TNF, cytokine/chemokine
	AKAP2	A kinase anchor protein 2	GPCR
	ANXA2	Annexin A2	Calcium, Wnt, NFκB, IL, EGFR, STAT3
	CXCL12	C-X-C motif chemokine ligand 12	GPCR, NFκB, chemokine
	GCSAM	Germinal center associated signaling and motility	B cell receptor
	GNAI2	G protein subunit alpha i2	GPCR, MAPK, chemokine
	GPS1	G protein pathway suppressor 1	GPCR, MAPK, JNK
	IFNGR2	Interferon gamma receptor 2	IFNγ, JAK-STAT
	IL2RG	Interleukin 2 receptor subunit gamma	IL, MAPK, PI3K/Akt, FGFR
	KRAS	KRAS proto-oncogene, GTPase	EGFR, MAPK, NFκB, Ras, Rac
	LGALS1	Galectin 1	IKK/NFκB
	PDE1B	Phosphodiesterase 1B	GPCR, PLC, FGFR, EGFR
	PPP3R1	Protein phosphatase 3 regulatory subunit B, alpha	MAPK, Wnt
	TLE4	Transducin like enhancer of split 4	Wnt, Notch
TRP47	BPI	Bactericidal/permeability-increasing protein	IL, TNF, TLR
	CAB39	Calcium binding protein 39	PI3K/Akt/mTOR, IGF1R,
	CDK1	Cyclin dependent kinase 1	p53, MAPK, Hedgehog
	CDK10	Cyclin dependent kinase 10	MAPK
	FYN	FYN proto-oncogene, Src family tyrosine kinase	Fcγ receptor, T cell receptor, MAPK, IKK/NFκB, PI3, C-type lectin receptor, VEGF receptor
	GNB1	G protein subunit beta 1	GPCR, Ras, Wnt, PI3K/Akt, Hedgehog, CXCR3/4, cytokine
	IGLL1	Immunoglobulin lambda like polypeptide 1	B cell receptor
	PRTN3	Proteinase 3	Cytokine, IL
	PTPN2	Protein tyrosine phosphatase, non-receptor type 2	ERK1/2, EGF receptor, IFNγ, IL, TNF, IFN, T cell receptor
TRP32	CD14	CD14 molecule	IKK/NFκB, MAPK, TLR, LPS
	CD63	CD63 molecule	Integrin, VEGF
	RC3H1	Ring finger and CCCH-type domain 1	NFκB, T cell receptor
TRP120/TRP47	IGKC	Immunoglobulin kappa constant	B cell receptor, MAPK, NFκB
TRP120/TRP32	IGHA1	Immunoglobulin heavy constant alpha 1	B cell receptor
	IGLL5	Immunoglobulin lambda like polypeptide 5	B cell receptor

An important host cellular process in which many TRP-interacting host proteins are involved is vesicle trafficking and intracellular transport. This process involves 20 TRP120-interacting proteins, seven TRP47-interacting proteins, and three TRP32-interacting proteins. Remarkably, from this group we found a subset of actin-related proteins interacting with *Ehrlichia* TRPs (particularly TRP120), including eight TRP120-interacting proteins and one TRP47-interacting protein (Table 5). *Ehrlichia* TRPs interact directly with host cytosolic actin gamma 1 (ACTG1) and eight other actin-binding or related proteins, including actin related protein 2/3 complex subunit 2 (ARPC2), CDC42 small effector 2 (CDC42SE2), integrin subunit alpha M (ITGAM), myosin X (MYO10), spectrin alpha erythrocytic 1 (SPTA1), WAS protein family member 2 (WASF2), WD repeat domain 1 (WDR1), and adenylate cyclase associated protein 1 (CAP1), suggesting that actin cytoskeleton plays an important role in ehrlichial infection. Most of these TRP-interacting proteins have also been found to be involved in phagocytosis or endocytosis. Multiple studies have shown the importance of TRP120 in ehrlichial binding and internalization, and most recently, we demonstrated that TRPs directly activate Wnt signaling to induce ehrlichial phagocytosis (Popov et al., 2000; Kumagai et al., 2010; Luo et al., 2016). In addition, it has been reported that *E. chaffeensis* is transported through the filopodia of the host cell and inhibition of actin polymerization in infected cells prevents filopodia formation. Moreover, the spreading process of *Ehrlichia canis* in macrophages is also dependent on actin cytoskeleton (Thomas et al., 2010; Alves et al., 2014). Other studies reported that *E. chaffeensis* surface protein EtpE binds GPI-anchored protein DNase X to trigger entry by N-WASP-dependent actin polymerization (Mohan Kumar et al., 2013, 2015). Therefore, interaction of TRPs with actin cytoskeleton might facilitate ehrlichial entry/release and necessary intracellular trafficking during *Ehrlichia* infection. Our RNAi data showed that knockdown of all these *Ehrlichia* TRP target proteins except WDR1 significantly reduced bacterial load, demonstrating the importance of actin cytoskeleton organization in ehrlichial entry and/or survival.

**Table 5:** *****E. chaffeensis*** TRP-interacting host proteins with primary function in actin cytoskeleton organization**.

**TRP**	**Host protein**		**Function**
	**Symbol**	**Full name**	
TRP120	ACTG1	Actin gamma 1	Component of the cytoskeleton and mediator of internal cell motility
	ARPC2	Actin related protein 2/3 complex subunit 2	Control of actin polymerization, phagocytosis and endocytosis, vesicle transport, membrane trafficking
	CDC42SE2	CDC42 small effector 2	F-actin accumulation, phagocytosis, regulation of cell shape
	ITGAM	Integrin subunit alpha M	Regulation of actin cytoskeleton, phagocytosis
	MYO10	Myosin X	Actin-based molecular motor, integration of F-actin and microtubule cytoskeletons, phagocytosis, intracellular transport, regulation of cell shape
	SPTA1	Spectrin alpha, erythrocytic 1	Actin binding and crosslinking, determination of cell shape, molecular scaffold organization of organelles
	WASF2	WAS protein family member 2	Actin binding, cytoskeleton organization, phagocytosis
	WDR1	WD repeat domain 1	Actin filament binding and fragmentation, disassembly of actin filaments
TRP47	CAP1	Adenylate cyclase associated protein 1	Actin polymerization or depolymerization, cell morphogenesis and polarity, endocytosis

Another group of TRP-interacting proteins with significant interest is host proteins involved in transcriptional regulation, including 16 TRP120-interacting proteins, seven TRP47-interacting proteins, and two TRP32-interacting proteins (Table 6). These proteins are either transcription factors or transcription regulators (coactivator or corepressor). Both TRP120 and TRP47 interact with PCGF5, a component of the polycomb repressive complex which mediates epigenetic regulation (Gao et al., 2014). Other important TRP120-interacting proteins involved in transcriptional regulation include AT-rich interactive domain 1B (ARID1B), cyclin-dependent kinase 12 (CDK12), interleukin enhancer binding factor 3 (ILF3), IRF2BP2, KDM6B, nuclear receptor binding SET domain protein 1 (NSD1), and tripartite motif containing 24 (TRIM24). Important TRP47-interacting proteins involved in transcriptional regulation include AT-rich interactive domain 2 (ARID2), histone deacetylase 2 (HDAC2), signal transducer and activator of transcription 5A and 6 (STAT5A and STAT6), and transcription factor EC (TFEC). Moreover, TRP32 interacts with DAZ-associated protein 2 (DAZAP2) and hematopoietically expressed homeobox (HHEX). We have previously reported that *Ehrlichia* TRP120 and TRP32 are nucleomodulins that directly bind host DNA and regulate the expression of numerous genes involved with transcriptional regulation, signal transduction, and cell differentiation and proliferation (Zhu et al., 2011; Farris et al., 2016). In this study, our RNAi data showed that knockdown of all *Ehrlichia* TRP target proteins involved in transcriptional regulation significantly influenced infection level, therefore, TRPs also play roles in regulation of host gene transcription by direct interactions with transcription factors and regulators in multiple cellular processes including survival, growth, and differentiation.

**Table 6:** *****E. chaffeensis*** TRP-interacting host proteins involved in transcriptional regulation**.

**TRP**	**Host protein**		**Main function**
	**Symbol**	**Full name**	
TRP120	ARID1B	AT-rich interactive domain 1B	A component of the SWI/SNF chromatin remodeling complex and may play a role in cell cycle activation
	CDK12	Cyclin-dependent kinase 12	Transcriptional regulation of genomic stability, cell cycle and cell differentiation
	ILF3	Interleukin enhancer binding factor 3	A double-stranded RNA-binding protein that forms a heterodimer with transcription factor ILF2 required for T-cell expression of interleukin 2
	IRF2BP	Interferon regulatory factor 2 binding protein 2	An interferon regulatory factor-2 (IRF2) binding protein that interacts with the C-terminal transcriptional repression domain of IRF2
	KDM6B	Lysine demethylase 6B	A histone H3-K27 demethylase involved in positive regulation of transcription
	NSD1	Nuclear receptor binding SET domain protein 1	A histone methyltransferase which may act as a nucleus-localized, basic transcriptional factor and also as a bifunctional transcriptional regulator
	TRIM24	Tripartite motif containing 24	A member of the tripartite motif family and mediates transcriptional control by interaction with the activation function 2 region of several nuclear receptors
TRP47	ARID2	AT-rich interactive domain 2	A subunit of the PBAF chromatin-remodeling complex which facilitates ligand-dependent transcriptional activation by nuclear receptors
	HDAC2	Histone deacetylase 2	Responsible for the deacetylation of lysine residues at the N-terminal regions of core histones and forms transcriptional repressor complexes by associating with many different proteins
	STAT5A	Signal transducer and activator of transcription 5A	A member of the STAT family of transcription factors
	STAT6	Signal transducer and activator of transcription 6	A member of the STAT family of transcription factors
	TFEC	Transcription factor EC	A member of the micropthalmia family of basic helix-loop-helix leucine zipper transcription factors which regulate the expression of target genes by binding to E-box recognition sequences
TRP32	DAZAP2	DAZ-associated protein 2	A coactivator of transcription factor TCF4 in canonical Wnt pathway
	HHEX	Hematopoietically expressed homeobox	Hematopoietic cell differentiation
TRP120/TRP47	PCGF5	Polycomb group ring finger 5	A component of the polycomb repressive complex which mediates epigenetic regulation

TRP-interacting targets include some host proteins involved in protein posttranslational modification (PTM). In particular, TRP120 interacts with 10 host proteins that have the main function in posttranslational modification machinery, including phosphorylation and ubiquitination. TRP120 interacts with host kinase and phosphatase involved in protein phosphorylation/dephosphorylation, including BMP2 inducible kinase (BMP2K), CDC-like kinase 1 (CLK1), and protein phosphatase 6 regulatory subunit 1 (PPP6R1). TRP120 also interacts with multiple host proteins involved in protein ubiquitination, including BTB domain containing 6 (BTBD6), a putative ubiquitin ligase adaptor protein (Sobieszczuk et al., 2010); cullin-associated and neddylation dissociated 1 (CAND1), an essential regulator of Cullin-RING ubiquitin ligases (Goldenberg et al., 2004); FBXW7, one of the four subunits of ubiquitin protein ligase complex SCFs (SKP1-cullin-F-box) (Hao et al., 2007); kelch-like family member 12 (KLHL12), a substrate adaptor of the Cullin-3 ubiquitin ligase complex (Jin et al., 2012); OTU deubiquitinase, ubiquitin aldehyde binding 1 (OTUB1), a highly specific ubiquitin iso-peptidase (Edelmann et al., 2009); and an ubiquitin precursor protein ubiquitin C (UBC). Ubiquitin B (UBB), another ubiquitin precursor, is a common interacting target of TRP120 and TRP47. TRP47 also interacts with NEDD4 binding protein 1 (N4BP1), an inhibitor of the E3 ubiquitin ligase (Oberst et al., 2007). Moreover, TRP120 has recently been found to be directly modified by SUMO, and more importantly, SUMO conjugation contributes to interactions with defined host proteins, such as PCGF5, actin and myosin cytoskeleton components, and recruitments of host proteins to the ehrlichial vacuole that influence infection (Dunphy et al., 2014). It remains unclear whether the functional consequences of TRP ubiquitination and phosphorylation are different from those associated with TRP SUMOylation, but it is well-established that many bacterial effectors mimic host proteins involved in the host posttranslational machinery to modify host proteins and signaling (Ribet and Cossart, 2010). Thus, PTMs may contribute to the moonlighting function of *Ehrlichia* TRPs and facilitate many diverse interactions between TRPs and host proteins. Another intracellular bacterium moonlighting effector is *Chlamydia trachomatis* CPAF, a protease which targets multiple host and bacterial proteins to maintain vacuole integrity, manipulate signaling pathways and promote virulence (Jorgensen et al., 2011; Zhong, 2011). Our RNAi data demonstrate that knockdown of all TRP target proteins involved in PTM significantly influenced infection, indicating that *E. chaffeensis* exploits major posttranslational modification machineries of host cells to facilitate a survival strategy.

Host proteins involved in metabolism are also frequent interacting targets of *E. chaffeensis* TRPs, including 16 TRP120-interacting proteins, six TRP47-interacting proteins, and five TRP32-interacting proteins. This category covers a variety of host metabolism processes, including glycometabolism, lipid metabolism, protein metabolism, nucleic acid metabolism, ion, and other inorganic metabolism. Two proteins, carbonic anhydrase I (CA1) and Charcot-Leyden crystal galectin (CLC) are common interacting targets of TRP120 and TRP47, suggesting their importance during infection. Our RNAi data demonstrated that knockdown of most of these *Ehrlichia* TRP target proteins significantly influenced bacterial load, indicating that TRPs also play a role in regulating host metabolism to favor the survival.

Interestingly, Y2H studies also discovered that a large number of apoptosis-associated proteins of the host cell were TRP targets, although most of these proteins are classified into other categories of function because their primary functions appear not to be related to apoptosis (Table 7). Two TRP32-interacting proteins glucocorticoid-induced 1 (GLCCI1) and tumor protein p53 inducible protein 11 (TP53I11) have main function in cell apoptosis. Expression of GLCCI1 is induced by glucocorticoids and may be an early marker for glucocorticoid-induced apoptosis (Chapman et al., 1996). TP53I11 is a downstream target of p53 and is involved in the regulation of apoptosis (Wu et al., 2009). Three common interacting proteins of TRPs, eukaryotic elongation factor 1 alpha 1 (EEF1A1), CA1, and CLC, play roles in apoptosis (Ejiri, 2002; Kubach et al., 2007; Zheng et al., 2015). In addition, TRPs interact with a wide variety of immunoglobulin molecules, such as IGKC, immunoglobulin heavy constant alpha 1 (IGHA1) and immunoglobulin lambda like polypeptide 5 (IGLL5), which have also been linked to apoptosis (Yang et al., 2009). TRP47 interacts with other seven host proteins associated with apoptosis, for example, STAT5A and STAT6, both of which are members of the STAT family of transcription factors and have been found to induce the expression of anti-apoptotic protein BCL2L1/BCL-XL (Calò et al., 2003); and CAP1, which has been implicated in promoting apoptosis by functioning as an actin shuttle to mitochondria (Wang et al., 2008). TRP120 interacts with relatively more apoptosis-associated proteins of the host cell. For example, CXCL12 is a negative regulator of intrinsic apoptotic signaling pathway in response to DNA damage, and FBXW7, in contrast, is a positive regulator of oxidative stress-induced intrinsic apoptotic signaling pathway (Hattermann et al., 2010). Previous studies have demonstrated that host cell apoptosis is delayed by stabilization of the mitochondrial membrane potential during *E. chaffeensis* and *E. ewingii* infection (Xiong et al., 2008; Liu et al., 2011). Our RNAi data showed that knockdown of all apoptosis-associated TRP target proteins (except for DEAD-box helicase 5 [DDX5]) significantly influenced bacterial load, thus, the interactions of TRPs with different apoptosis-associated proteins may serve an opposite function by inhibiting or promoting apoptosis in the different stages of infection, to facilitate ehrlichial survival and release, respectively.

**Table 7:** *****E. chaffeensis*** TRP-interacting host proteins involved in cell apoptosis**.

**TRP**	**Host protein**		**Main function**
	**Symbol**	**Full name**	
TRP120	ICAM3	Intercellular adhesion molecule 3	Vesicle trafficking
	SPTA1	Spectrin alpha, erythrocytic 1	
	ADAM17	ADAM metallopeptidase domain 17	Cell signaling
	CXCL12	C-X-C motif chemokine ligand 12	
	KRAS	KRAS proto-oncogene, GTPase	
	LGALS1	Galectin 1	
	PDE1B	Phosphodiesterase 1B	
	PPP3R1	Protein phosphatase 3 regulatory subunit B, alpha	
	DDX5	DEAD-box helicase 5	Transcriptional regulation
	IRF2BP	Interferon regulatory factor 2 binding protein 2	
	KDM6B	Lysine demethylase 6B	
	TRIM24	Tripartite motif containing 24	
	CAT	Catalase	Metabolism
	FBXW7	F-box and WD repeat domain containing 7	Posttranslational modification
	ERAL1	Era-like 12S mitochondrial rRNA chaperone 1	Others
	ORAOV1	Oral cancer overexpressed 1	
	SEPX1	Selenoprotein X, 1	
TRP47	CDK1	Cyclin dependent kinase 1	Cell signaling
	GNB1	G protein subunit beta 1	
	PTPN2	Protein tyrosine phosphatase, non-receptor type 2	
	HDAC2	Histone deacetylase 2	Transcriptional regulation
	STAT5A	Signal transducer and activator of transcription 5A	
	STAT6	Signal transducer and activator of transcription 6	
	CAP1	Adenylate cyclase associated protein 1	Vesicle trafficking and intracellular transport
TRP32	CD14	CD14 molecule	Cell signaling
	GLCCI1	Glucocorticoid-induced 1	Apoptosis
	TP53I11	Tumor protein p53 inducible protein 11	
TRP120/TRP47	CA1	Carbonic anhydrase 1	Metabolism
	CLC	Charcot-Leyden crystal galectin	
	IGKC	Immunoglobulin kappa constant	Cell signaling
TRP120/TRP32	EEF1A1	Eukaryotic translation elongation factor 1 alpha 1	Others
	IGHA1	Immunoglobulin heavy constant alpha 1	Cell signaling
	IGLL5	Immunoglobulin lambda like polypeptide 5	

We classified TRP-interacting host proteins according to their main functions, but many of them are moonlighting proteins which could be included in multiple categories. For example, EEF1A1, a common target protein of TRP120 and TRP32, is the second most abundant protein in eukaryotes after actin. It is an isoform of the alpha subunit of the elongation factor-1 complex, so its main function is the enzymatic delivery of aminoacyl tRNAs to the ribosome during protein translation; however, EEF1A1 is also one of the most important multifunctional eukaryotic proteins and has been reported to be involved in regulation of transcription, cytoskeletal remodeling, cellular response to epidermal growth factor stimulus, regulation of chaperone-mediated autophagy, and apoptosis (Condeelis, 1995; Ejiri, 2002). IGKC, another common target protein of TRP120 and TRP47, is the immunoglobulin kappa constant domain, so its main function is immune response including antigen binding, immunoglobulin receptor binding and complement activation, but it also appears to be involved in other signaling pathways, vesicle-mediated transport, receptor-mediated endocytosis, and phagocytosis (Bentley and Rabbitts, 1980). It is not clear if all or part of the functions of these proteins are involved in *Ehrlichia* infection, but some of them are very likely moonlighting when interacting with TRPs, since they belong to multiple important common categories of TRP-interacting proteins, such as cell signaling, transcriptional regulation, vesicle trafficking and apoptosis. In addition, some putative host proteins interact with two TRPs, including EF1A1, IGHA1, IGLL5 (interacting with both TRP32 and TRP120), PCGF5, IGKC, CA1, CLC, and UBB (with TRP47 and TRP120), implicating the importance of not only the moonlighting and overlapping host targets but also the crosstalking and converging cellular networks by *Ehrlichia* effectors.

Our siRNA experiments revealed that knockdown of TRP-interacting host proteins could either increase or decrease *E. chaffeensis* load. Most siRNAs decreased the bacterial load, indicating that these proteins are necessary for *Ehrlichia* infection; however, siRNAs of 18 TRP120-interacting proteins and one TRP47-interacting protein (PCGF5) increased the bacterial load, indicating that these proteins inhibit the *Ehrlichia* infection. For example, Golgi associated gamma adaptin ear containing ARF binding protein 1 (GGA1) is a ubiquitous coat protein that regulates the protein trafficking between the trans-Golgi network and the endosome/lysosome system (Bonifacino, 2004), so *E. chaffeensis* may inactivate GGA1 to prevent early endosomal maturation into late endosomes and then fusion with lysosome. PCGF5 is a component of the polycomb repressive complex which controls expression of many developmental regulator genes (Gao et al., 2014), thus, *E. chaffeensis* may inhibit this transcriptional repressor in order to modulate host cell gene expression to favor ehrlichial survival. Uniquely, knockdown of many TRP120 host targets promoted infection, suggesting that TRP120 plays a role in modulating levels of these targets during infection. This result is likely related to our recent finding that TRP120 can function as ubiquitin ligase and degrade host target proteins such as PCGF5 (Zhu and McBride unpublished). In this study, no siRNA of TRP32-interacting proteins was found to increase the bacterial load significantly, probably because only C-terminus of TRP32 was available as the bait of Y2H screening and resulted in the loss of some host proteins interacting with other domains of TRP32. In addition, siRNAs of 14 TRP-interacting proteins could not change bacterial load significantly. This is probably because these proteins are important at the late stage of *Ehrlichia* infection, or they play a nonessential role in infection. Some of these proteins may be too abundant to be reduced sufficiently, or they are just nonspecific Y2H targets. We did not perform the late time point of infection in our experiments, since some infected cells started to collapse and release *Ehrlichia* at 3 day p.i.

Our RNAi data also show that changes in *Ehrlichia* infection status can occur at different stages of infection for different host proteins, suggesting that *Ehrlichia* TRPs and host proteins interact at different time points during infection. For example, some siRNAs, such as those targeting CDC42SE2, ITGAM, MYO10, unc-13 homolog D (UNC13D) and TLE4, reduced bacterial load significantly at 2 days p.i. but not significantly at day 1, suggesting that these proteins may be not involved in bacterial internalization or early stage of infection. In contrast, some siRNAs, such as those targeting solute carrier family 2 member 3 (SLC2A3), interferon gamma receptor 2 (IFNGR2), PDE1B, ARID1B, and EIF3A, reduced or increased bacterial load significantly at 1 day p.i. but not significantly at day 2, suggesting that these proteins are more important for the bacterial internalization or early stage of infection. Meanwhile, most siRNAs reduced or increased bacterial load significantly at both 1 and 2 days p.i., suggesting that these proteins are important for both early and intermediate stages of infection. Moreover, knockdown of some TRP-interacting proteins had a dramatic effect on infection, suggesting that they play key roles. For example, knockdown of SPTA1, ILF3, CLC, UBB, SEPX1, and PCGF5 reduced or increase the infection very significantly at both 1 and 2 days p.i., suggesting that they are critical factors exploited by *Ehrlichia* TRPs for bacterial survival; however, we cannot exclude the possibility that siRNAs may have different knockdown efficiency. We also found that the knockdown of any single target protein by RNA interference could not abolish the ehrlichial growth completely. As observed by Western blot, 100% knockdown of a target protein by siRNA is very difficult, thus, a knockout cell line would be necessary to determine if a host protein is essential for *E. chaffeensis* infection.

The molecular mechanisms by which *Ehrlichia* enters and survives in the host cell remain unclear. This study documents the extensive effector-host interactions that contribute to survival of this obligately intracellular pathogen, and will help understand the molecular mechanisms utilized by other intracellular pathogens to infect host cells. Further studies will delineate the specific roles of these complex and diverse TRP-host interactions during infection.

## Author contributions

TL and PD designed, performed the experiments and analyzed the data. TL wrote the manuscript. JM directed and contributed to the writing of the manuscript.

## Funding

This work was supported by grants AI106859 and AI126144 from the National Institute of Allergy and Infectious Diseases (NIAID), and by funding from the Clayton Foundation for Research (to JM).

### Conflict of interest statement

The authors declare that the research was conducted in the absence of any commercial or financial relationships that could be construed as a potential conflict of interest.
